# Glioblastoma cancer stem cell lines express functional acid sensing ion channels ASIC1a and ASIC3

**DOI:** 10.1038/s41598-017-13666-9

**Published:** 2017-10-20

**Authors:** Yuemin Tian, Pia Bresenitz, Anna Reska, Laila El Moussaoui, Christoph Patrick Beier, Stefan Gründer

**Affiliations:** 1 Institute of Physiology, RWTH Aachen University, Pauwelsstrasse 30, D-52074 Aachen, Germany; 2 Department of Neurology, RWTH Aachen University, Pauwelsstrasse 30, D-52074 Aachen, Germany; 3 Department of Neurology and Department of Clinical Research, Odense University Hospital and University of Southern Denmark, Sdr. Boulevard 20, 5000 Odense C, Denmark

## Abstract

Acidic microenvironment is commonly observed in tumour tissues, including glioblastoma (GBM), the most aggressive and lethal brain tumour in adults. Acid sensing ion channels (ASICs) are neuronal voltage-insensitive sodium channels, which are sensors of extracellular protons. Here we studied and functionally characterized ASICs in two primary glioblastoma stem cell lines as cell culture models. We detected transcripts of the *ACCN2* and *ACCN3* genes, coding for ASIC1 and ASIC3, respectively, but not transcripts of *ACCN1* (coding for ASIC2). Available microarray data confirmed that *ACCN1* is downregulated in glioma. Western blotting confirmed expression of ASIC1 and ASIC3, the most proton-sensitive ASICs, in both GBM cell lines. We characterized ASICs functionally using whole-cell patch clamp and detected different types of acid-sensitive currents. Some of these currents had kinetics typical for ASICs and were sensitive to specific toxin inhibitors of ASIC1a or ASIC3, demonstrating that the GBM cell lines express functional ASIC1a and ASIC3 that may enable GBM cells to sensitively detect extracellular pH in a tumour tissue. Microarray data revealed that expression of *ACCN2* and *ACCN3* is associated with improved survival of patients suffering from gliomas, suggesting that preserved susceptibility to extracellular pH may impair tumour growth.

## Introduction

Glioblastoma multiforme (GBM) is the most common malignant tumour of the brain, exhibiting high rates of proliferation and infiltration. Despite recent advances in understanding of its pathogenesis, GBM remains incurable and has a poor prognosis. GBM contain a rare subpopulation of cells with stem cell-like properties, so-called cancer stem cells or tumour-initiating cells, that differentiate into cells of all three neural lineages including cells expressing markers of immature neurons. GBM stem cell (GSC) lines show a remarkable degree of genetic stability^1^ and represent the best available *in vitro* model to study the biology of GBM. GSC lines also mimic the intertumoural molecular heterogeneity of human GBM, and GSC lines resembling all major molecular subtypes have been described, including GSC lines with proneural and mesenchymal expression patterns^2^.

Tumours have a high metabolic rate and often limited blood supply. Therefore, an acidosis is commonly observed in tumour tissues^3^ and associated with decreased proliferation of GSC lines *in vitro* ^4^ but may induce acquisition of stem cell properties^5^. Acid-sensing ion channels (ASICs) are ligand-gated cation channels that are directly activated by extracellular protons^6^. They are quickly activated by acidic transients and desensitize in the continued presence of protons^7^. They are expressed in virtually every neuron^6^ and in some glia cells^8^ and therefore represent candidate proton sensors also in GBM tissue. ASICs assemble as homo- or heterotrimers^9,10^. In the brain, ASICs are mainly composed of homomeric ASIC1a or heteromeric ASIC1a/2a and ASIC1a/2b^11–14^. Homomeric ASIC1a is highly sensitive for protons with half-maximal activation at around pH 6.6^6,15^. Similarly sensitive is ASIC3, which in rodents is relatively specifically expressed in the peripheral nervous system^16^. In humans, however, ASIC3 is also expressed in the brain^17^.

Previously, it had been found that cell lines derived from malignant glioma express an amiloride-sensitive constitutive Na^+^ conductance that is not detected in normal astrocytes or low grade gliomas^18,19^. It has been reported that this cation conductance is due to an unconventional assembly of ASIC and epithelial sodium channel (ENaC) subunits^20^ and that it regulates migration and cell cycle progression in gliomas^21^. Besides this non-canonical channel, the expression of ASICs has not been investigated in GBM. Moreover, ASIC expression has so far not been investigated in GSC lines containing cell populations that do grow in non-clonal spheres and better represent tumour tissue than clonal cell lines^1^.

Here we molecularly and functionally characterized ASICs in two established GSC lines – R54, a CD133^+^, proneural-like GSC line, and R8, a CD133^−^, mesenchymal-like GSC line^22^. We show that R54 and R8 GSC cell lines express functional ASIC1a and ASIC3 that may serve as sensitive sensors of extracellular pH, and available microarray data suggest that expression of ASICs is associated with an improved survival.

## Results

### The GSC lines R54 and R8 express ASIC1a and ASIC3 mRNA and protein

**Figure 1:**
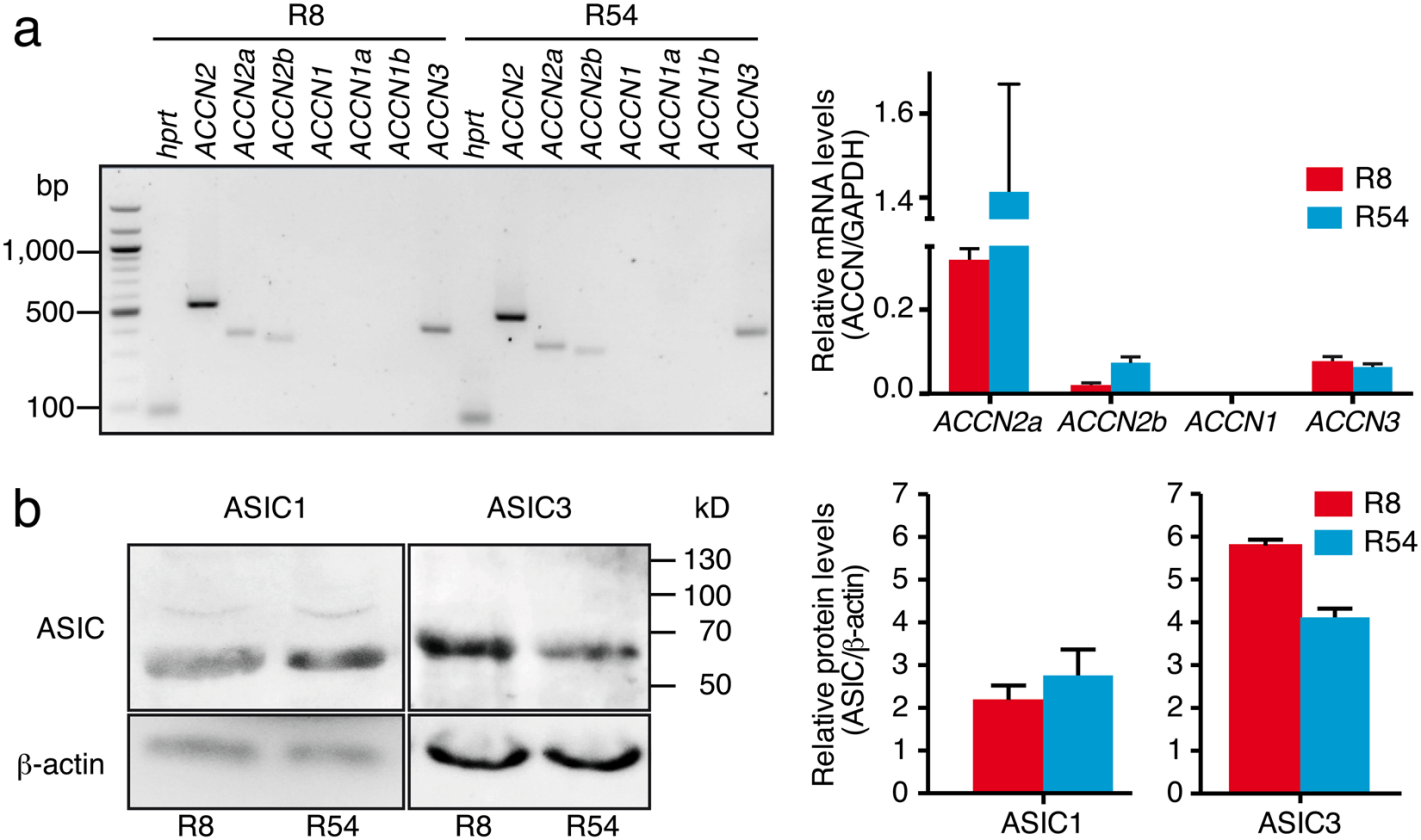
ASIC1 and ASIC3 are expressed in GSC lines. (**a**) Left, RT-PCR analysis revealed expression of *ACCN2a*, *ACCN2b* and *ACCN3* but not *ACCN1* in R8 and R54 cells respectively. HPRT (hypoxanthine-guanine phosphoribosyltransferase) served as a reference gene. Right, qPCR analysis revealed different expression levels of *ACCN2a*, *ACCN2b* and *ACCN3* in R8 and R54 cells. GAPDH (glyceraldehyde 3-phosphate dehydrogenase) served as a reference gene. (**b**) Left, western blot analysis revealed expression of ASIC1 and ASIC3 in GSC lines, ß-actin was used as control. Right, summary of four Western blots for ASIC1 and three Western blots for ASIC3; expression was normalized to ß-actin.

To account for the molecular heterogeneity of GBM, we chose a GSC line with a proneural expression pattern (R54) and a GSC line with a more mesenchymal expression pattern (R8). First, we examined by RT-PCR the expression of the genes that contribute to proton-gated ASICs (ASIC1a, ASIC1b, ASIC2a, ASIC2b and ASIC3) in R54 and R8 cells. The *ACCN2* gene (amiloride-sensitive cation channel neuronal 2) codes for ASIC1a and ASIC1b, *ACCN1* for ASIC2a and ASIC2b and *ACCN3* for ASIC3, respectively. We detected transcripts of *ACCN2* variant a (ASIC1a) and b (ASIC1b) and of *ACCN3* (ASIC3), but not of ACCN1 (ASIC2a and ASIC2b), in R54 and R8 cells (Fig. 1a, left). We quantified mRNA expression of *ACCN* genes by quantitative RT-PCR (Fig. 1a, right). While *ACCN2* variant a showed the highest expression in both R54 and R8 cells, it was approximately 4.5-fold higher in R54 than R8. *ACCN3* expression was lower but comparable in R54 and R8 cells, such that the ratio of *ACCN2*a/*ACCN3* was different in the two GSC cells lines, being ~20 in R54 and ~4 in R8 cells. In whole brain, ASIC1a mRNA is approximately 7-fold more abundant than ASIC3 mRNA^17^. Thus, compared to whole brain the ratio of *ACCN2*a/*ACCN3* is increased in R8 and decreased in R54 cells. *ACCN2* variant b (ASIC1b) was weakly expressed in R54 and very weakly expressed in R8. Abundance of *ACCN2* variant b was about 5% that of variant a in R54 cells and 6% in R8 cells. Thus, compared to whole brain, where abundance of *ACCN2* variant b is about 2% that of variant a^23^, expression of variant b relative to variant a was slightly increased in the GSC lines, but still low. Real time PCR confirmed the absence of *ACCN1* (ASIC2) transcripts in R54 and R8 cells (Fig. 1a, right).

To confirm the expression of ASIC1 and ASIC3 in GSC lines, we extracted proteins from R54 and R8 cells and subjected them to Western blot analysis with ASIC1- and ASIC3- specific antibodies, revealing the presence of ASIC1 and ASIC3 in both R8 and R54. While the protein abundance of ASIC1a was comparable in both cell lines, ASIC3 was significantly more abundant in R8 than in R54 cells (n = 3, *P = *0.006, unpaired t-test; Fig. 1b). Thus, PCR and Western blot analysis showed that R54 and R8 cells express various ASICs, mainly ASIC1a and ASIC3, but also ASIC1b, with ASIC3 being more abundant in R8 than R54 cells.

### Functional characterization of ASICs in proneural-like CD133^+^ R54 cells

**Figure 2:**
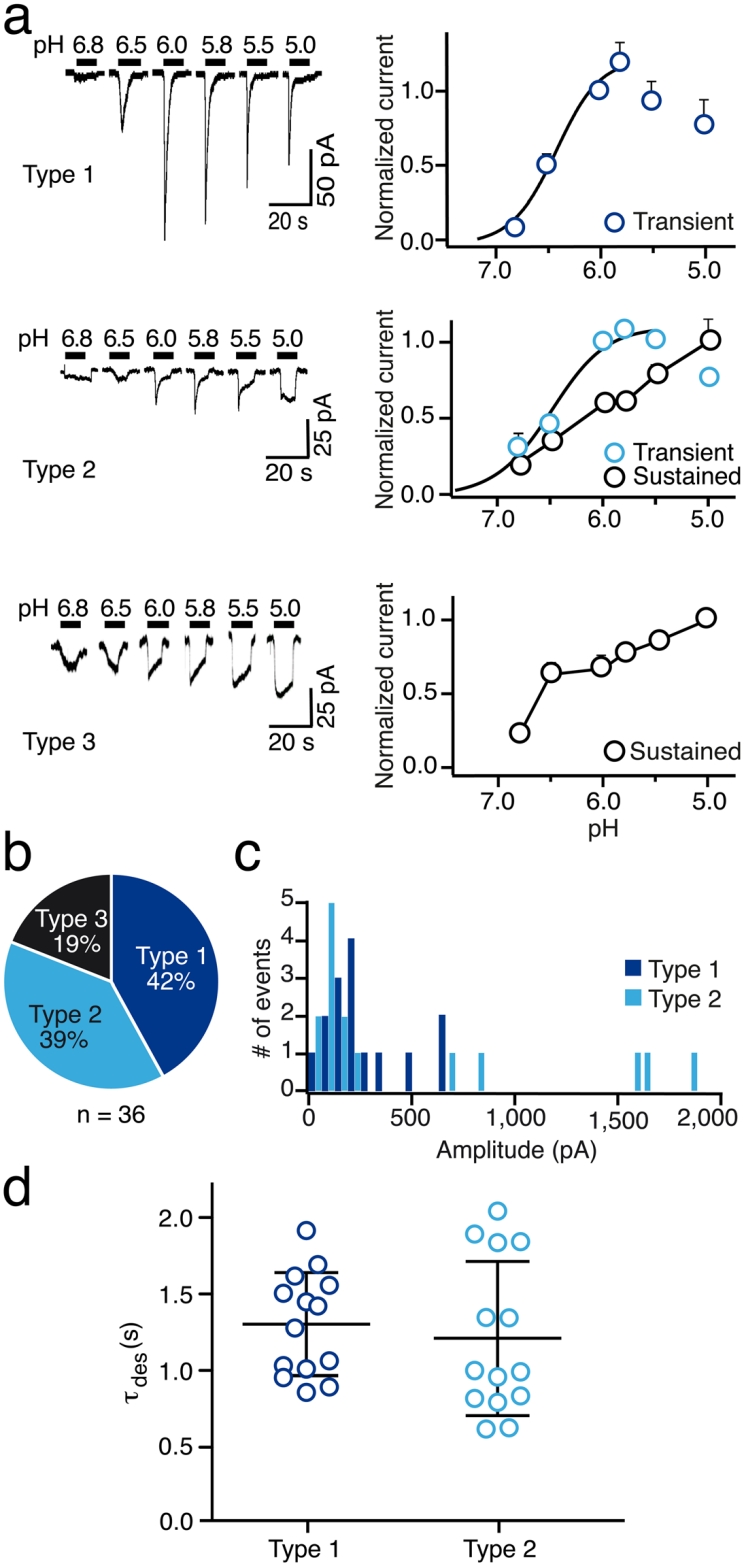
Electrophysiological characterization of ASICs in R54 cells. (**a**) Left, whole cell current traces representative for type 1, type 2, and type 3 currents, activated by different acidic pH; conditioning pH was 7.4. Right, pH-response relationships. Peak currents from type 1 and type 2 cells were normalized to the current at pH 6.0. At pH ≥ 6.5, in most cases there was no transient current, and the amplitude of the inward current upon acidification was measured. The curves represent fits to the Hill equation. Amplitudes of sustained currents from type 2 and type 3 cells were determined just before stepping back to pH 7.4 and were connected by lines. (**b**) Pie chart illustrating the relative occurrence of type 1, type 2, and type 3 currents. (**c**) Frequency histogram of peak amplitude of type 1 (dark blue) and type 2 (light blue) transient currents. (**d**) Time constants of desensitization τ_des_ of transient type 1 and type 2 currents.

We then functionally characterized ASICs in GSC lines by whole cell patch clamp. R54 cells responded to a rapid change of bath pH from 7.4 to pH 6.8 or below with one of three kinetic patterns (Fig. 2a). Almost half of the cells (42%) responded with a typical transient ASIC inward current (type 1). A similar fraction of cells (39%) responded with a biphasic current, composed of a fast transient current and a sustained component that did not fully inactivate while the pH remained acidic (type 2). Relatively few cells (19%) had no transient current and responded with a small sustained inward current (type 3).

Robust transient currents were elicited by pH ≤ 6.5. Transient current amplitudes started to saturate around pH 6.0 and could be fitted with a Hill equation (Fig. 2a), revealing half-maximal activation of type 1 currents at pH 6.4 ± 0.1 (n = 8) and of type 2 currents at pH 6.5 ± 0.1 (n = 7), consistent with the presence of highly proton-sensitive ASIC1a or ASIC3. In all cells, the amplitudes of transient type 1 and type 2 currents decreased with repeated application of more acidic solutions (Fig. 2a). Such a tachyphylaxis is unique to homomeric ASIC1a^24,25^, suggesting the presence of ASIC1a in these cells. In contrast to transient currents, sustained currents (type 2 and type 3) did not saturate but linearly increased with decreasing pH. We did not investigate sustained currents further.

Figure 2c shows the distribution of transient peak amplitudes at pH 6.0. In most cells the transient currents had peak amplitudes between 50–200 pA for both type 1 and 2, but in some cells type 2 currents were huge with amplitudes >1,000 pA. Sustained currents of type 3 had maximal amplitudes <100 pA. Desensitization time constant τ_des_ at pH 6.0 was 1.4 ± 0.1 sec for type 1 currents (n = 15) and 1.2 ± 0.1 sec for transient type 2 currents (n = 14; Fig. 2d), which are typical for ASIC1a but slower than desensitization of ASIC3.

**Figure 3:**
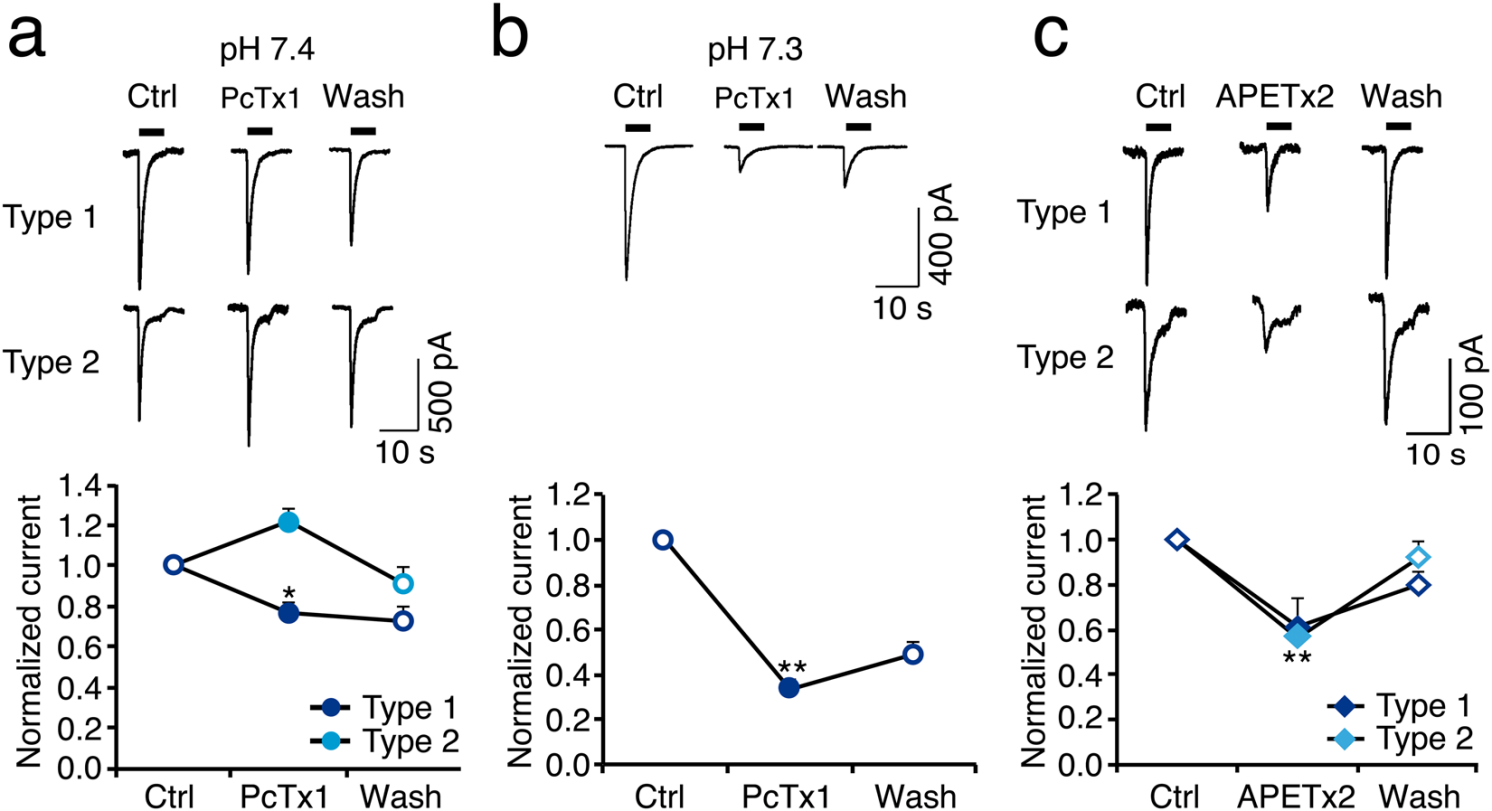
Toxin sensitivity of ASICs in R54 cells. (**a**) Top, representative whole cell current traces for type 1 and type 2 currents, activated by pH 6, before, during and after application of 30 nM PcTx1; conditioning pH was 7.4. Bottom, summary of peak currents before, during and after PcTx1. n = 4. (**b**) As in (a) but with conditioning pH 7.3. n = 5. (**c**) As in (a) but with 500 nM APETx2. n = 5. *P < 0.05; **P < 0.01.

So far, our results are consistent with the presence of ASIC1a and ASIC3 in R54 cells. These two ASICs can be differentiated by their sensitivity to animal toxins, PcTx1 and APETx2. PcTx1 potently inhibits homomeric ASIC1a and potentiates ASIC1b but has not effect on ASIC3^26–28^. APETx2 inhibits ASIC3 but not ASIC1a^29^. Heteromeric ASIC1a/ASIC3 are insensitive to PcTx1 and are inhibited by APETx1 with a lower potency than homomeric ASIC3^26,29^. At conditioning pH 7.4, pre-application of PcTx1 for two min only slightly inhibited type 1 currents, while it potentiated type 2 currents (n = 4 for each; Fig. 3a), suggesting the presence of ASIC1b in some R54 cells^28^. Inhibition by PcTx1 is pH-sensitive, however^27^, and steady-state desensitization of human ASIC1a is slightly shifted to more acidic pH compared to rat ASIC1a^30^. Indeed, at conditioning pH 7.3, PcTx1 strongly inhibited ASIC currents in R54 cells (n = 5; Fig. 3b), indicating a high abundance of homomeric ASIC1a. The inhibition by PcTx1 was only partially reversible, probably due to tachyphylaxis of the ASIC1a current^24^. Tachyphylaxis may slightly overestimate the inhibition by PcTx1. Pre-application of APETx2 (500 nM) for one min substantially reduced transient type 1 and 2 currents by approximately 40% (n = 5 and 6, respectively; Fig. 3c), demonstrating a considerable contribution of ASIC3 to ASIC currents in most R54 cells. In summary, the functional properties of proton-gated currents in R54 cells suggest the predominant presence of homomeric ASIC1a in these cells plus ASIC3-containing ASICs, which could be either heteromeric ASIC1a/3 or homomeric ASIC3.

**Figure 4:**
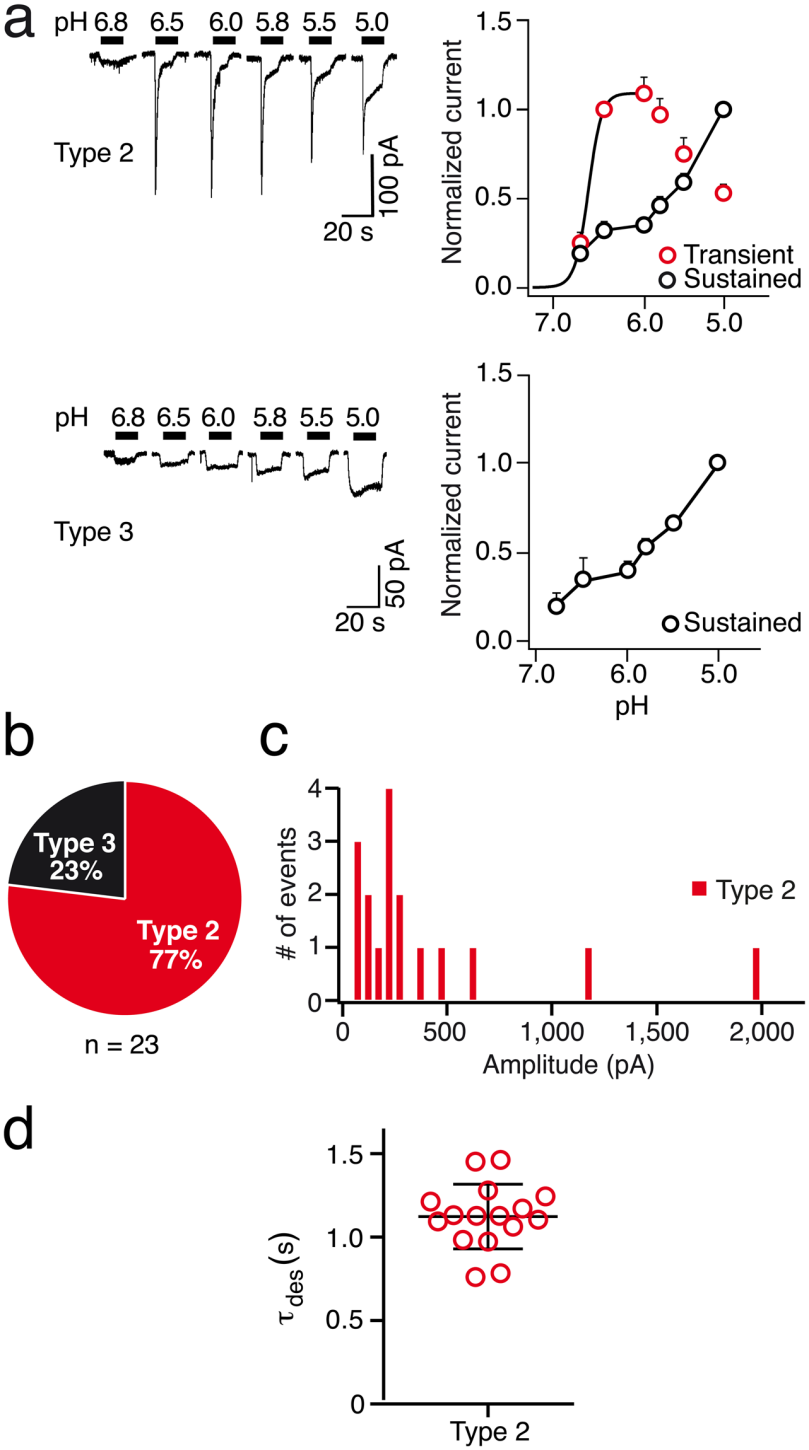
Electrophysiological characterization of ASICs in R8 cells. (**a**) Left, whole cell current traces representative for type 2 and type 3 currents, activated by different acidic pH; conditioning pH was 7.4. Right, pH-response relationships. Peak currents from type 2 cells were normalized to the current at pH 6.5. The curves represent fits to the Hill equation. Amplitudes of sustained currents from type 2 and type 3 cells were normalized to pH 5 and were connected by lines. (**b**) Pie chart illustrating the relative occurrence of type 2 and type 3 currents. (**c**) Frequency histogram of peak amplitude of type 2 transient currents. (**d**) Time constants of desensitization τ_des_ of transient type 2 currents.

### Functional characterization of ASICs in mesenchymal-like CD133^−^ R8 cells

R8 cells did not have type 1 currents. As shown in Fig. 4, most cells (77%) had biphasic currents with a transient peak current and a sustained component (type 2). The remaining cells (23%) had sustained currents without any transient component. Transient currents could be fitted with a Hill equation. They saturated as pH was reduced with a half-maximal response at pH 6.7 ± 0.1 (n = 10; Fig. 4a), again revealing the presence of a highly proton-sensitive ASIC. In all cells, transient currents also displayed tachyphylaxis, suggesting the presence of homomeric ASIC1a. In contrast, sustained current amplitudes increased linearly as the pH was lowered to 5.0.

**Figure 5:**
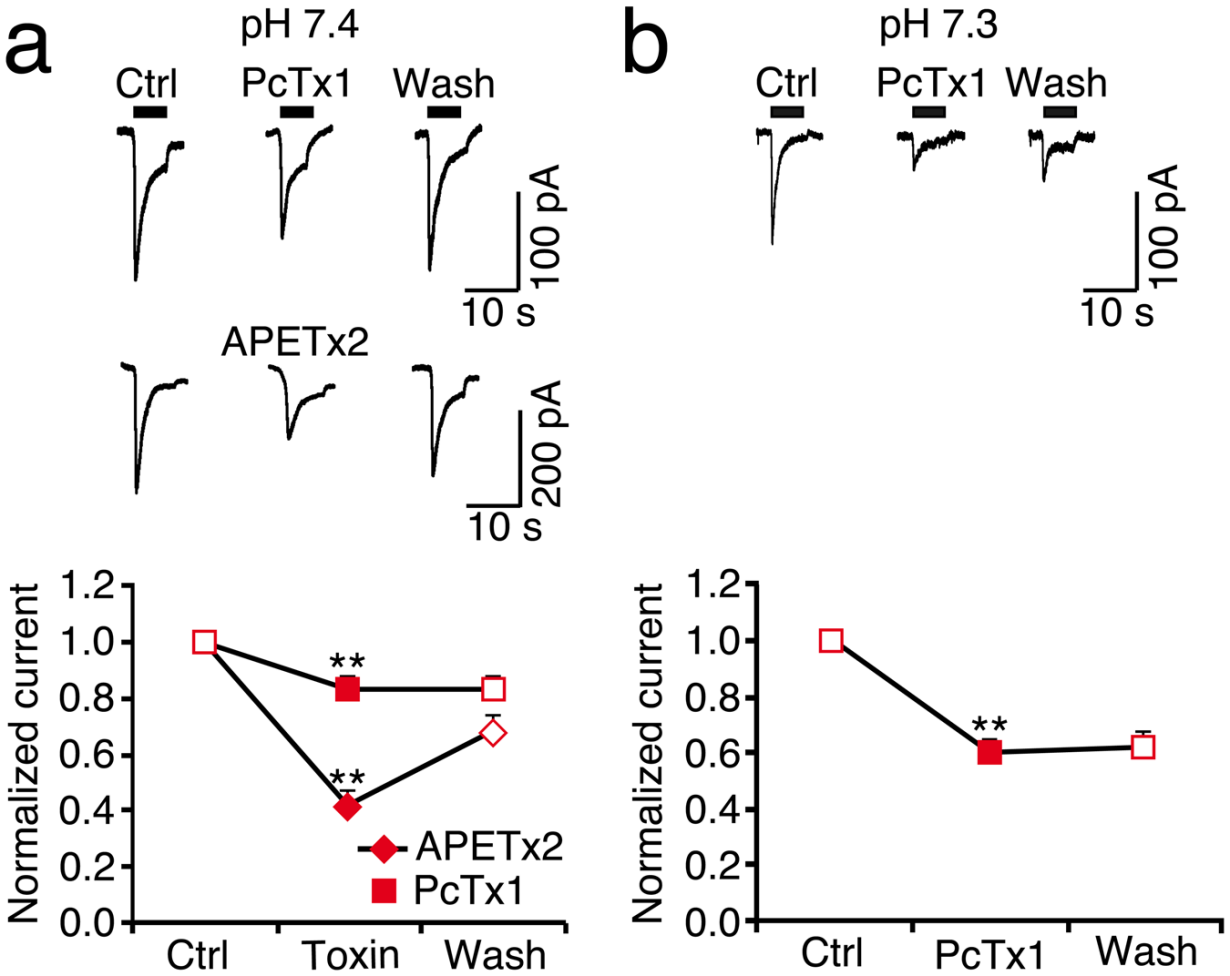
Toxin sensitivity of ASICs in R8 cells. (**a**) Top, representative whole cell current traces for type 2 currents, activated by pH 6, before, during and after application of either 30 nM PcTx1 or 500 nM APETx2, respectively; conditioning pH was 7.4. Bottom, summary of peak currents before, during and after toxin application. n = 10 for PcTx1, n = 8 for APETx2. (**b**) As in (a) but with conditioning pH 7.3. n = 8, **P < 0.01.

In most cells the transient currents had peak amplitudes between 50–400 pA (Fig. 4c). Desensitization time constant τ_des_ at pH 6.0 of transient type 2 currents was 1.2 ± 0.1 sec (n = 17), suggesting the presence of homomeric ASIC1a. (Fig. 4d). The transient component of type 2 currents in R8 cells was slightly sensitive to pre-application of PcTx1 for two min at conditioning pH 7.4 (16% inhibition; n = 5; Fig. 5a), but more sensitive at conditioning pH 7.3 (57% inhibition; n = 8; Fig. 5b). It was also sensitive to pre-application of APETx2 for one min (57% inhibition) (n = 8; Fig. 5c). These results are similar to R54 cells and are compatible with the presence of both homomeric ASIC1a and ASIC3-containing channels, which could be either homomeric ASIC3 or heteromeric ASIC1a/3.

**Figure 6:**
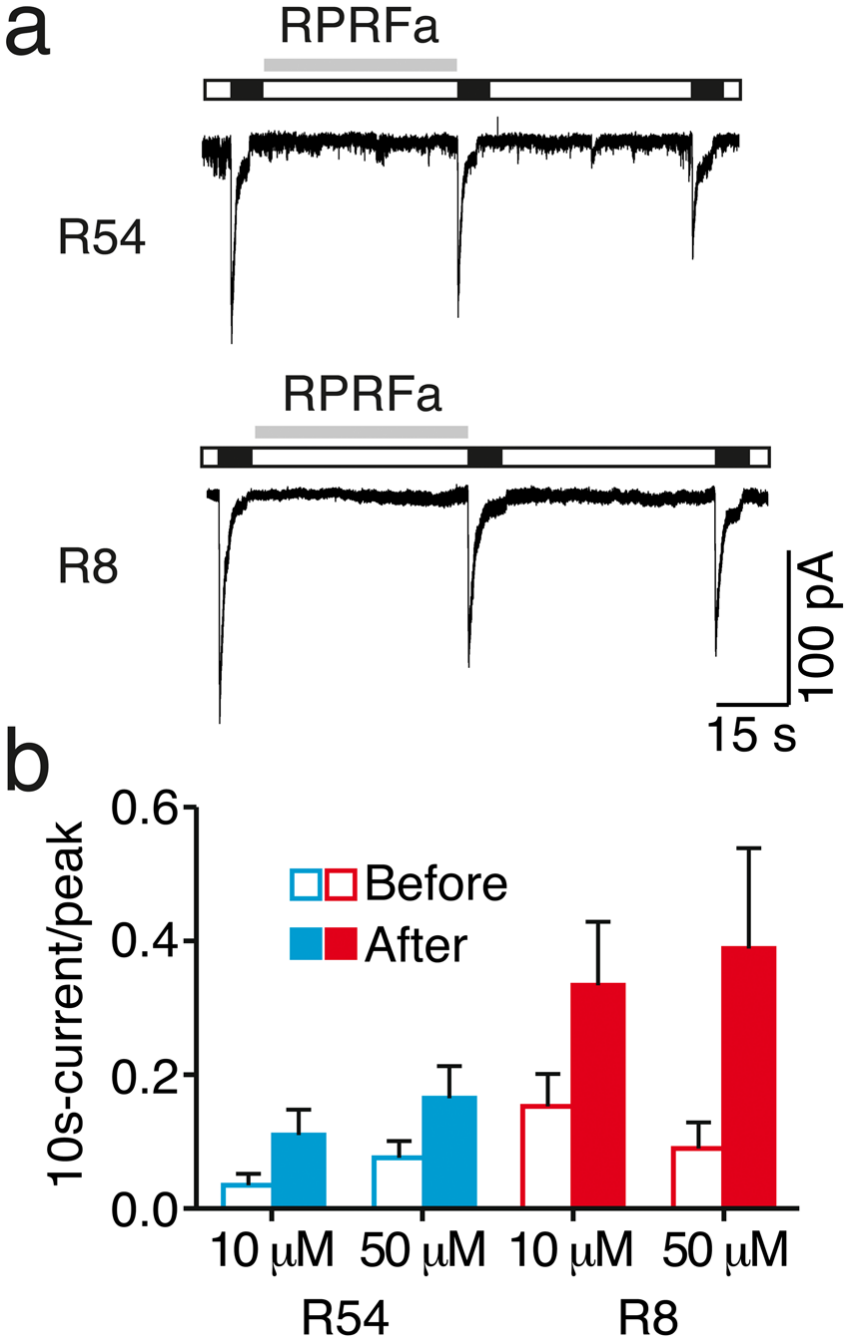
RPRFa potentiates ASIC-like currents in GSC lines. (**a**) Representative current traces from R54 and R8 cells, showing currents before and after pre-application of 50 μM RPRFa. (**b**) Analysis of the current 10 secs after activation, normalized to the peak current amplitude, reveals a slight potentiation by RPRFa. n = 5 for R54, n = 4 for R8; *P* > 0.05.

We tried to obtain further evidence for the presence of ASIC3-containing channels in R54 and R8 cells and pre-applied a recently discovered cone snail neuropeptide^31^, RPRFa, that potentiates currents of ASIC3-containing channels but not of homomeric ASIC1a^31^. The main effect of RPRFa is a slowing of the desensitization. Pre-application of 10 μM or 50 μM RPRFa indeed slowed desensitization of ASIC currents in R54 and R8 cells, as judged by the current remaining after 10 secs normalized to the peak current amplitude (n = 5, *P* = 0.06 for R54; and n = 4, *P* = 0.12 for R8; Fig. 6). This result provides further evidence for the presence of functional ASIC3 in both R54 and R8 cells.

**Figure 7:**
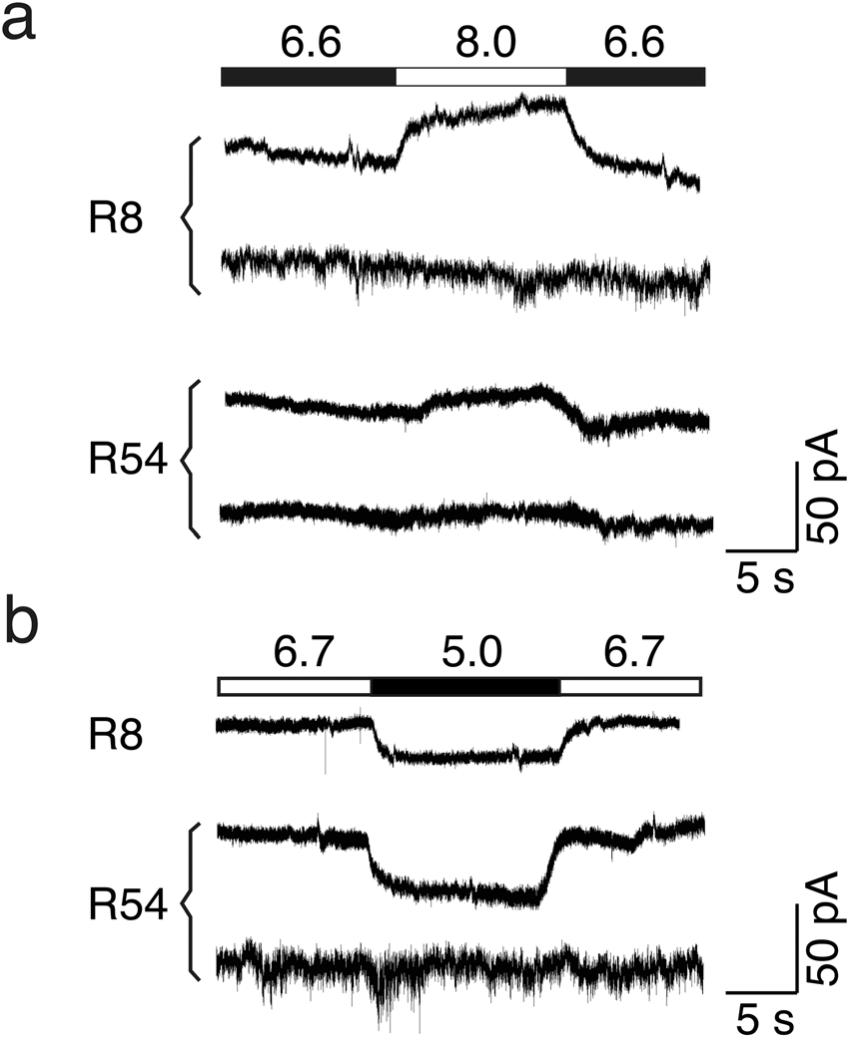
There is no alkalization-sensitive ASIC3 current and no ASIC1b current in R54 or R8 cells. (**a**) Current traces from R54 and R8 cells, showing either no current when stepping from pH 6.6 to pH 8.0 or a small outward current. Traces are representative for 4 cells, each. Stepping from acidic pH 6.6 to alkaline pH 8.0 did never elicit inward currents. (**b**) Current traces from R54 and R8 cells, showing either no current when stepping from conditioning pH 6.7 to pH 5.0 or a small sustained inward current. Traces are representative for 8 cells (R8) or 4 cells (R54), respectively. Stepping from pH 6.7 to pH 5.0 did not elicit typical transient ASIC currents.

It has been reported that human ASIC3 has the unique property to sense not only acidification but also alkalization; this property is intrinsic to homomeric ASIC3 and heteromeric ASIC1a/3^17^. So far, however, it has only been reported for human ASIC3 in heterologous expression systems. Therefore, we addressed the question whether endogenous ASICs in GBMs have the capacity to sense alkalization by stepping from acidic pH 6.6 to alkaline pH 8.0. Neither in R54 nor in 8 R8 cells did we detect inward currents when going from acidic to more alkaline pH (n = 8 for each cell cline; Fig. 7a). Also during our characterization of ASICs in R54 and R8 cells (see above), did we observe only a single R8 cell that showed an inward current when we stepped back from pH 6.0 to conditioning pH 7.4. This current was not APETx-sensitive, however, and therefore probably not carried by ASIC3^17^. Thus, we found no evidence for the capacity of ASICs in GBMs to sense alkalization.

*ACCN2* variant b (ASIC1b) was also weakly expressed in R54 and R8 cells (Fig. 1a). We used a conditioning pH 6.7, at which ASIC1a and ASIC3 but not ASIC1b are desensitized^23,30,32^, to detect hASIC1b currents. However, under these conditions application of pH 5.0 did not elicit transient ASIC-like current, neither in R54 nor R8 cells (Fig. 7b). Thus, expression of functional ASIC1b in R54 and R8 cells was too weak to be detected functionally.

### Cells within GSC lines have no amiloride-sensitive constitutive current

**Figure 8:**
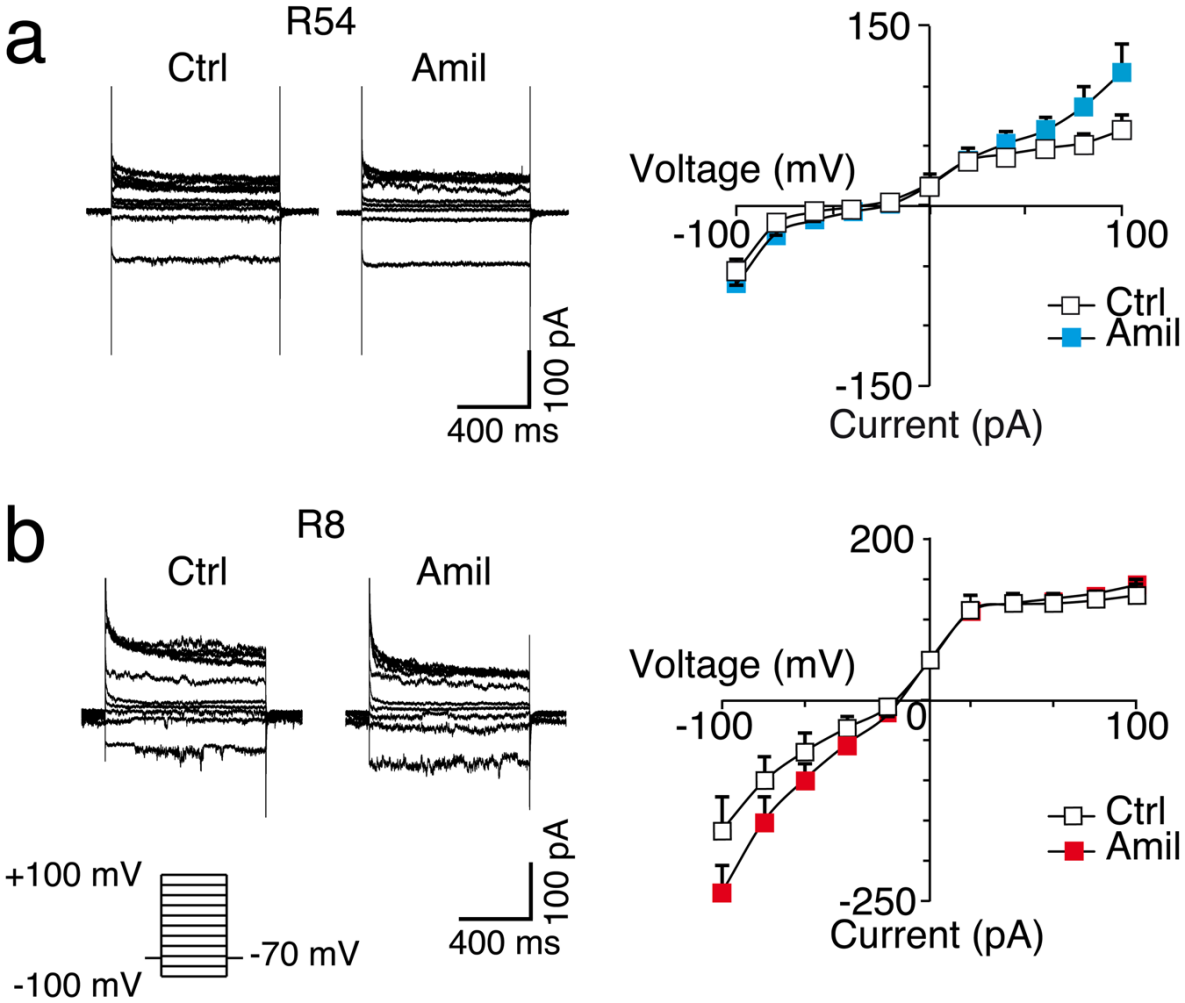
There is no constitutive amiloride-sensitive Na^+^ conductance in R54 or R8 cells. (**a**) Left, representative whole cell current traces from R54 cells without and with amiloride. Cells were clamped in 20 mV steps for 800 ms to holding potentials ranging from −100 to +100 mV. Right, I/V curves do not reveal a decrease of the conductance by amiloride. n = 6. (**b**) as in (a) but for R8 cells. n = 6.

It has been reported that primary cultures obtained from fresh brain tumour tissue as well as established clonal cell lines that were originally derived from human glioblastoma have a constitutive conductance that is sensitive to amiloride^18,19^ and that is presumably formed by a DEG/ENaC with unconventional composition, containing ASIC1a together with subunits of the epithelial Na^+^ channel^20^. To test whether R54 and R8 cells have a similar amiloride-sensitive basal current, we performed whole cell patch clamp experiments in which we changed the holding potential from −100 mV to +100 mV in 20 mV steps in the presence and absence of 100 μM amiloride. If anything, the basal conductance of R54 and R8 cells was increased in the presence of amiloride (*P* = 0.075 for R54 and *P* = 0.053 for R8, paired t-test; Fig. 8), indicating that GSC lines have no unconventional amiloride-sensitive constitutive conductance.

### Ca^2+^ imaging revealed the heterogeneity of GSC lines

As expected, our electrophysiological analysis revealed that cells within GSC lines are heterogeneous. To quickly gain a more comprehensive picture of the heterogeneity of GSC lines, we performed Ca^2+^ imaging. It has previously been reported that TRPV1 is highly expressed in brain tumour^33^ and that TRPM8 is expressed in a human glioblastoma cell line^34^; both appear to be upregulated in patients diagnosed with glioblastoma^35^. We therefore examined whether pH 6.0, the TRPV1 agonist capsaicin, the TRPM8 agonist menthol or ATP were able to elicit a Ca^2+^ response in R54 and R8 cells. Moreover, cells were depolarized by 30 mM K^+^ to test whether they have voltage-gated Ca^2+^ channels (VGCCs). Ionomycin was used as positive control.

**Figure 9:**
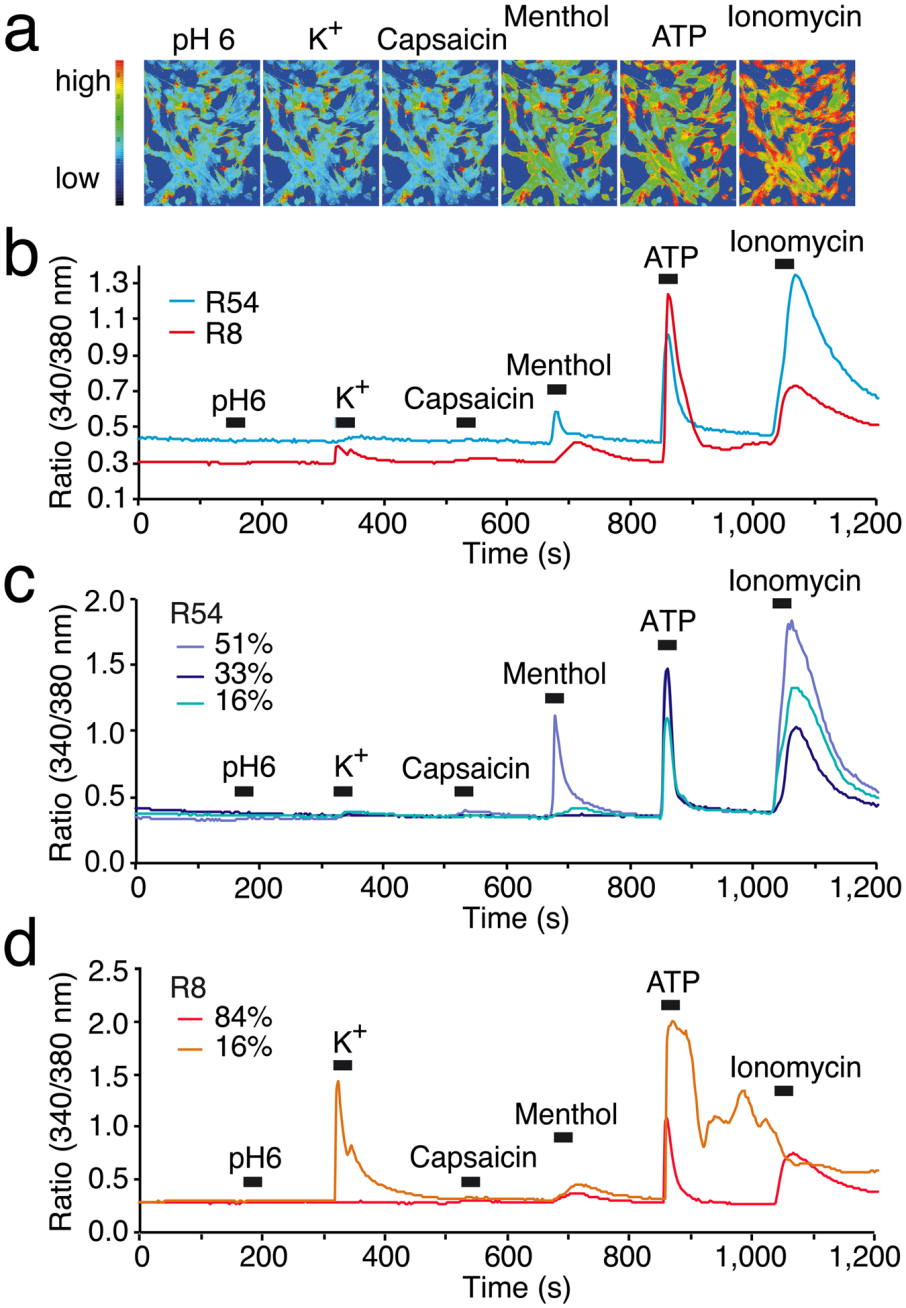
Ca^2+^ responses of GSC lines to different stimulations. (**a**) Pseudo-coloured images of representative experiments in R54 cells showing relative levels of Ca^2+^ concentration (blue = low; red = high), as visualized by Fura-2 340/380 nm fluorescence. Different stimuli are indicated. (**b**) Mean Ca^2+^ responses of 97 R54 (blue line) and 61 R8 cells (red line) to stimulation by pH 6, 30 mM K^+^, 100 μM capsaicin, 200 μM menthol, 100 μM ATP, or 1 μM ionomycin, respectively. (**c**) The population of R54 cells was divided according to whether they quickly responded to menthol with a Ca^2+^ peak (49 cells; marine blue line) or slowly with a Ca^2+^ plateau (16 cells; light blue line), or whether they did not respond to menthol (32 cells; dark blue line). (**d**) The population of R8 cells was divided according to whether they responded to K^+^ (10 cells; orange line) or not (51 cells; red line).

The cells of GSC lines indeed showed different responses to the same stimulations. Figure 9a shows examples of pseudo-coloured images of Ca^2+^ signals during applications of different compounds to R54 cells and Fig. 9b summarizes the mean responses of 61 R8 cells and 97 R54 cells. pH 6 elicited a slight Ca^2+^ response in only 1–2% of the cells from R54 and R8. Since Ca^2+^ permeability of ASICs is low^36^, cells need to have VGCCs that are activated by the depolarization due to ASIC activation, to secondarily raise the Ca^2+^ concentration after pH 6 stimulation. Indeed, none of the 97 R54 cells responded with a Ca^2+^ increase by a depolarization with high K^+^, suggesting that R54 cells are not excitable. In contrast, 16% of the R8 cells showed various increases in intracellular Ca^2+^ concentration in high K^+^, showing that R8 cells contain a relatively small fraction of excitable cells. As these cells did not respond to pH 6 with a Ca^2+^ signal, they either did not express an ASIC at sufficiently high amounts (perhaps type 3 currents) or the depolarization by ASIC activation was not strong enough to activate VGCCs. Capsaicin did not elicit any Ca^2+^ response, neither in R54 nor in R8 cells, suggesting that they do not express TRPV1. In strong contrast, menthol elicited in 67% of the R54 cells and in all R8 cells a Ca^2+^ response, showing that the majority of R54 and all R8 cells express TRPM8. 100 μM ATP elicited a Ca^2+^ response in 97% of R54 cells and in 95% R8 cells, demonstrating that they either express P2Y or P2X receptors or both. The results from calcium imaging are summarized in Table 1.

**Table 1:** Ca^2+^ responses in R54 and R8 cells.

	R54	R8
pH 6	1% positive	2% positive
30 mM K^+^	0% positive	16% positive
capsaicin	0% positive	0% positive
menthol	67% positive	100% positive
ATP	97% positive	95% positive
ionomycin	100% positive	100% positive

Percentages of cells responding with an increase in intracellular Ca^2+^ concentration are indicated. Total number of cells was 97 for R54 and 61 for R8.

### Expression of *ACCN2* or *ACCN3* is associated with improved survival

**Figure 10:**
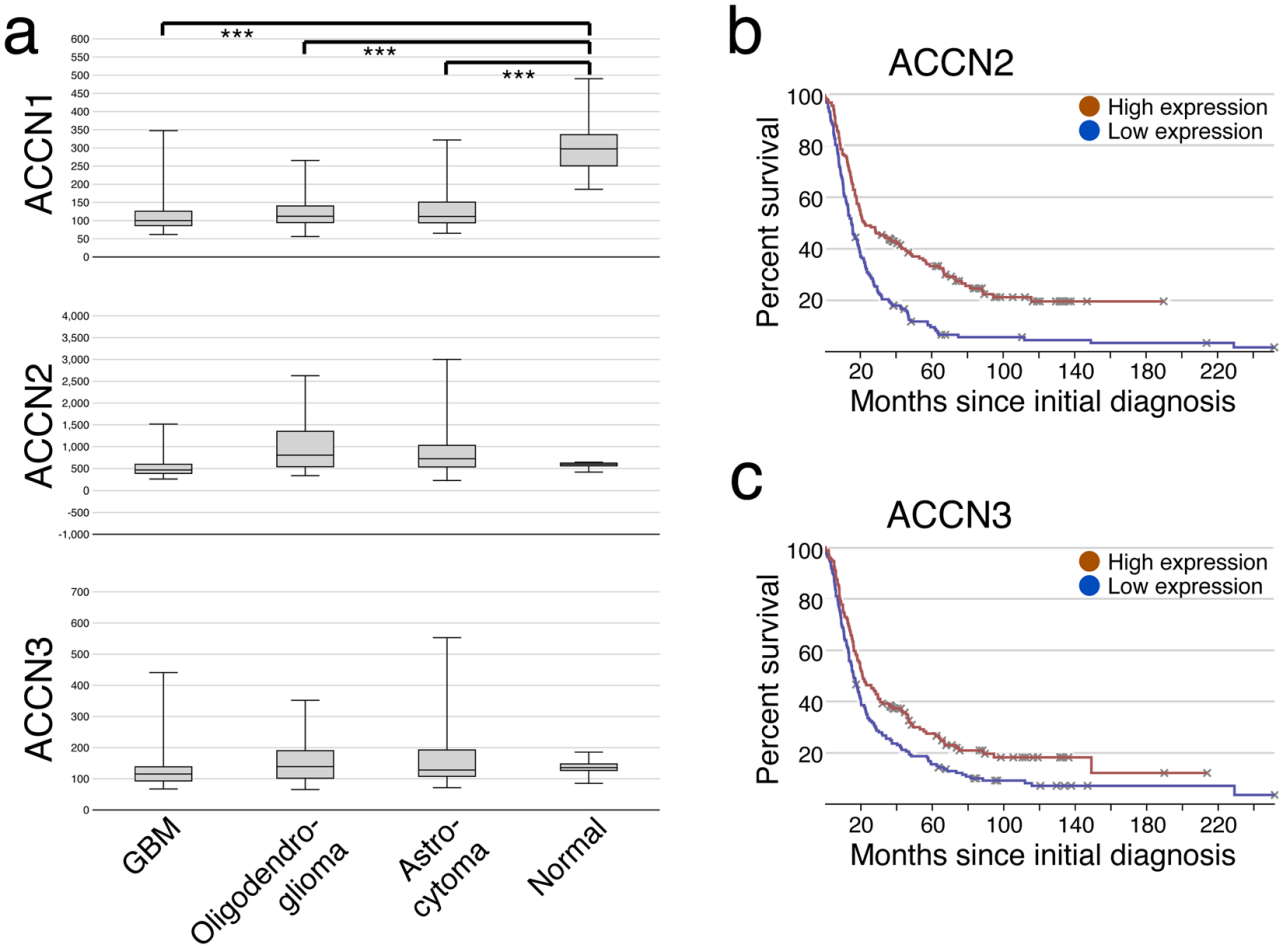
Expression of *ACCN1-3* and Kaplan-Meier survival analyses. All 329 glioma patients with available mRNA data from the Rembrandt database were used to determine the relative expression of *ACCN1-3* in human glioma and normal brain and the association of *ACCN2* and *ACCN3* expression with overall survival. **(a)** Boxplots illustrating the expression level of *ACCN1* (ASIC2, top; p < 0.01, Student´s t-test), *ACCN2* (ASIC1, centre), and *ACCN3* (ASIC3, bottom) in GBM, oligodendroglioma, astrocytoma, and non-tumour control, respectively. Whiskers display the minimal and maximal values, respectively. ***P < 0.001. **(b**,**c)** Kaplan-Meier survival curves of pooled glioma samples (including GBM, oligodendroglioma, and astrocytoma) with high or low expression (above or below the median) of *ACCN2* or *ACCN3*. **(b)** Survival depending on high or low *ACCN2* expression (P < 0.001, log-rank test). **(c)** Survival depending on high or low *ACCN3* expression (P = 0.003, log-rank test).

To confirm the expression of ASICs in human GBM samples, we analysed the expression levels in Repository of Molecular Brain Neoplasia Data (Rembrandt), a cancer clinical genomics database and a Web-based data mining and analysis platform, established by the National Cancer Institute^37^. Rembrandt contains data from different glioma subtypes, including glioblastoma multiforme (WHO grade IV), astrocytoma (WHO grade II), and oligodendroglioma (WHO grade II). In line with our *in vitro* results, *ACCN1* (ASIC2) was downregulated in all types of glioma samples as compared to the non-tumour samples used as control (*P* < 0.001, two-sided t-test; Fig. 10a). In contrast, *ACCN2* and *ACCN3* showed similar expression levels as compared to non-tumour groups irrespective of tumour grade (Fig. 10a). The Kaplan-Meier analyses unveiled a significant survival benefit of glioma patients with high *ACCN2* (*P* < 0.001, log-rank test; Fig. 10b) or high *ACCN3* expression (*P* = 0.003, log-rank test; Fig. 10c).

## Discussion

Our results show the expression of ASICs in two molecularly distinct GSC lines . We detected mRNA and protein expression for ASIC1a and ASIC3 (Fig. 1), the two most proton-sensitive ASICs^7^. We did not find a substantial expression of ASIC2 in the GSC lines studied and inspection of published microarray data^37^ revealed a significant downregulation in tumour samples as compared to non-tumour samples. This supports the previously described anti-migratory and therefore anti-oncogenic effects of ASIC2 in glioma cells^38^. The expression of either *ACCN2* or *ACCN3* was associated with a significant survival benefit in glioma samples, underscoring their clinical relevance. Electrophysiological characterization revealed clear evidence for the presence of functional homomeric ASIC1a and homo- and/or heteromeric ASIC3-containing ASICs (summarized in Table 2), demonstrating that the *ACCN2* and *ACCN3* mRNA is also translated in GSC cell lines. Tachyphylaxis of transient ASIC currents in most cells indicated the presence of homomeric ASIC1a^24,25^, the half-maximal activation around pH 6.5 is characteristic for both ASIC1a^6,15^ and ASIC3^16,32^, and the sensitivity of most currents to PcTx1 or APETx1 is indicative of homomeric ASIC1a and ASIC3-containing channels, respectively^26,29^. Time constants of desensitization τ_des_ in R54 cells were more similar to ASIC1a (Table 2), but showed a rather large range, compatible with mixed populations of ASIC1a and other more rapidly desensitizing ASICs. In R8 cells, τ_des_ was more homogenous and similar to ASIC1a (Table 2), but was still compatible with mixed populations of ASIC1a and other ASICs. The slight potentiation of ASIC currents in R54 and R8 cells by the cono-RFamide RPRFa also indicates the presence of ASIC3-containing channels in these cells^31^. Thus, to our knowledge, this is the first time that ASIC3 was characterized in native human cells. This is the more important as it is generally considered to be a specific receptor of the peripheral nervous system.

**Table 2:** Functional properties of ASIC currents in R54 and R8 cells compared to properties of recombinant human ASICs in heterologous expression systems.

		τ_des_ (sec at pH 5)	pH50 act.
R54	Type 1	1.4 ± 0.1 (15)	6.4 ± 0.1 (8)
	Type 2	1.2 ± 0.1 (14)	6.5 ± 0.1 (7)
R8	Type 2	1.2 ± 0.1 (17)	6.7 ± 0.1 (10)
hASIC1a		1.1–3.0^1,2^	6.45–6.55^1,2^
hASIC1b		0.55^3^	5.9^3^
hASIC3		0.5 ± 0.04 (7) at pH 6	6.55^4^

Values for R54 and R8 cells are mean ± SEM; the number n of measurements is indicated in parentheses. Values for recombinant ASICs are from the literature; references are indicated in superscripts and are as follows: ^1^(Gunthorpe, 2001), ^2^(Sherwood, 2008), ^3^(Hoagland, 2010), ^4^(Delaunay, 2012). n.d., not determined. τ_des_ for hASIC3 was determined in HeLa cells with a recombinant clone.

We detected also low amounts of ASIC1b mRNA in R54 and R8 cells (Fig. 1a) but had no clear electrophysiological evidence for the presence of functional ASIC1b. ASIC1b is less sensitive to protons than either ASIC1a or ASIC3^15,23,39^ with a half-maximal activation at pH 5.9 (Table 2). Since transient currents saturated at pH 6.0, no substantial amount of ASIC1b was present in these cells. Only the slight potentiation of type 2 currents when PcTx1 was applied at a conditioning pH of 7.4 (Fig. 3a) suggested the presence of functional ASIC1b in these cells^28^.

Thus, it is clear that in both cell types, R54 and R8, homomeric ASIC1a and homo- and/or heteromeric ASIC3-containing ASICs were present. We obtained no unequivocal results concerning the relative ratios of these two channel populations, however. For example, in R54 cells at conditioning pH 7.3 > 60% of the cells were sensitive to PcTx1 (Fig. 3b), indicative of homomeric ASIC1a^26^, but ~60% of the cells were also sensitive to APETx1 (Fig. 3c), indicative of ASIC3-containing channels^29^. This discrepancy might be due to the heterogeneity of the GBM cells that we analysed. Moreover, although we avoided long-time passaging of the cells, we cannot completely exclude that the GBM cell population changed its composition over time. The presence and co-existence of ASIC1a and ASIC3 in both R54 and R8 cells was a consistent finding, however.

In Ca^2+^ imaging, only around 1% of cells responded to acidic pH (pH 6.0) with a calcium signal (Table 1) even though ~80% of the cells express ASICs (Figs 2 and 4). Although homomeric ASIC1a is known to be slightly permeable to Ca^2+^ ^6^, its Ca^2+^ permeability is low^36,40^. Thus, voltage-gated Ca^2+^ channels (VGCCs) are necessary to mediate a Ca^2+^ influx upon depolarization by ASIC activation. In agreement with the inability of pH 6.0 to produce an intracellular Ca^2+^ signal, only 16% of the R8 cells and none of the R54 cells responded to the strong depolarization induced by 30 mM K^+^ with a Ca^2+^ signal (Table 1), demonstrating that R54 cells do not express VGCCs and only a small subpopulation of R8 cells does so.

It has previously been reported that ASIC1 and ASIC2 are present in different cell lines derived from glioblastoma patients^18,19^. But rather than mediating typical transient ASIC currents, it was found that they assemble in a non-canonical mixed ASIC/ENaC channel that mediates a constitutive amiloride-sensitive Na^+^ conductance^20^. We found no evidence for such a conductance in GSC lines, however (Fig. 8), which may be due to the use of GSC lines cultured in serum-free medium that more closely mirror the phenotype and genotype of primary tumours than do serum-cultured cell lines^1^. We therefore conclude that the unconventional constitutive amiloride-sensitive Na^+^ conductance does at least not play a general role in GBMs.

Our data clearly show that glioma cells express functional ASIC1a and ASIC3, with substantial impact on patient survival. The molecular mechanisms behind the putative anti-oncogenic effects of ASICs expression is, however, unknown. Data from *in vivo* and *in vitro* studies clearly indicate that the average pH is ~0.2 pH units lower in GBM (pH 6.97) than in normal brain tissue (pH 7.2)^41^. Anti-angiogenic treatment with e.g. bevacicumab may further decrease pH. If low pH alone is beneficial or detrimental for glioma cells is currently unknown. *In vitro*, low pH impairs proliferation of GSC lines^4^, however low pH induces stem cell properties and GSC lines challenged with low pH become significantly more aggressive as compared to GSC lines cultured under standard condition^5^.

Although ASICs desensitize in the continued presence of protons^7^, it has been shown that ASIC3 can also detect sustained acidification in the pH range around 7.0, which might enable this channel to detect the acidification that accompanies heart ischemia^42^. In addition, human ASIC1a is less sensitive to protons than its rodent orthologues, such that it will not be desensitized at pH 7.0^30^. Thus, pH sensitivity of ASIC1a and ASIC3 might just be tuned such as to endow GBMs with the capacity to detect and signal the acidic environment surrounding it. One might speculate that this capacity increases the susceptibility of glioma cells to extracellular acidosis. Our data allow, however, no conclusion on the precise mechanisms involved. Moreover, ASICs may have different impact on different tumours, because breast cancer cells also express ASIC1 and alterations in *ACCN2* (including amplification, mutations and upregulation) are associated with poor prognosis^43^. In addition, ASIC1 and ASIC3 contribute to epithelial-mesenchymal transition of pancreatic cancer cells^44^. Future studies need to clarify the exact role of ASIC1 and ASIC3 in different tumours.

In summary, we identify and functionally characterize ASICs as sensitive proton sensors in glioma cells. ASICs endow glioma cells with the capacity to sense the tumour-surrounding pH, which may be associated with an improved prognosis.

## Methods

### Cell culture

GSC lines were grown, as previously described^2^, in DMEMF12 (Gibco) supplemented with 2% B27 supplement, 1% glutamine, 1% vitamin, 1% penicillin-streptomycin, 0.1% fibroblast growth factor (FGF) and 0.1% epidermal growth factor (EGF) at 37 °C in a humidified atmosphere with 5% CO_2_.

### Reverse transcription and quantitative real-time PCR

Total RNA was isolated from GSC lines using RNeasy minikit (Qiagen, Venlo, The Netherlands). Concentration and quality of the RNA was measured using a NanoDrop 2000c spectrophotometer (Thermo Scientific). RNAs with a 260 nm/280 nm ratio >2.00 and a 260 nm/230 nm ratio >1.80 were used for reverse transcription. First-strand cDNA was synthesized from 1 μg total RNA using QuantiTect Reverse Transcription Kit (Qiagen), yielding 20 μl cDNA. All kits were used according to manufacturer’s instructions. Contamination with genomic DNA was controlled by RT-PCR using intron-spanning primers for the reference gene hypoxanthine-phosphoribosyl-transferase (HPRT).

1 μl cDNA was used for each PCR reaction. Sequences of primers for standard PCR were as follows: hHPRT-sense, 5′-GGA CCC CAC GAA GTG TTG GAT ATA AG-3′, hHPRT-antisense, 5′-GTC AAG GGC ATA TCC TAC AAC AAA CTT G-3′; ASIC1-sense, 5′-CCT GCT CTG GAC TTC CTG-3′, ASIC1-antisense, 5′-CCT CGA ACG TGC CTC GGG-3′; ASIC1a-sense, 5′-GCC GGT GAG CAT CCA GGC-3′, ASIC1a-antisense, 5′-CCT GCA GTA TCT CCA GCT G-3′; ASIC1b-sense, 5′-CCA TCA CCA GCA GCA GGA C-3′, ASIC1b-antisense, 5′-GAC AGC CGC ACA GCA TTA G-3′; ASIC2-sense, 5′-CTG TTT ACA GCA TCA CCG-3′, ASIC2-antisense, 5′-CCA AGC AGG TCT AAT AGC-3′; ASIC2a-sense, 5′-GCC AAC ACC TCC ACC CTC-3′, ASIC2a-antisense, 5′-CCG GGA TCT GCA GGT TGA C-3′; ASIC2b-sense, 5′-CGC ATG GCC CGC GAG GAG-3′, ASIC2b-antisense, 5′-GCG GCT CCA CTC GCG GTG-3′; hASIC3-sense, 5′-CAA CAT CAA CCC ACT GCG C-3′, hASIC3-antisense, 5′-GTT TGA GGT GGG GAT CCG AG-3′.

For quantitative real-time PCR (qPCR), hydrolysis probes (TaqMan probes) for GAPDH (glyceraldehyde-3-phosphate dehydrogenase), ASIC1a, ASIC1b, ASIC2 and ASIC3 were ordered from Applied Biosystems (the assay identification numbers are Hs02758991_g1, Hs00952802_m1, ASIC1B, Hs00153756_m1, Hs00245097_m1). Each reaction, containing 1 µl cDNA, 1 µl TaqMan Gene Expression Assay and 5 µl 2x Rotor-Gene Probe PCR Master Mix (Qiagen), was performed in triplicates; a sample without cDNA served as negative control. qPCR was performed in a Rotor-Gene Q (Qiagen), starting with a long denaturation phase (10 min, 95°), followed by 40 cycles with denaturation (15 s, 95 °C) and annealing/elongation (60 s, 60 °C). Experiments were repeated with RNA from at least n = 3 independent cell batches and analysed using the ΔΔC_t_ method. Efficiency of each probe was determined by a standard curve and was close to 100%. Results are reported as relative levels of ASIC/GAPDH mRNA.

### Patch clamping

GSC lines were grown on laminin and ornithine coated coverslips as described^2^. Coverslips were mounted in a perfused bath on the stage of an inverted microscope (IX71, Olympus) and kept at room temperature. The bath solution contained (in mM): NaCl 128, KCl 5.4, HEPES 10, glucose 5.5, MgCl_2_ 1, CaCl_2_ 2; pH was adjusted to 7.4. For low pH bath solution, HEPES was replaced by MES, and the pH adjusted appropriately. Patch-clamp experiments were performed in the whole-cell configuration. Patch pipettes had an input resistance of 4–6 MΩ, when filled with an intracellular-like solution containing (in mM): NaCl 10, KCl 121, HEPES 10, EGTA 5, MgCl_2_ 2; pH was adjusted to 7.2. Currents were recorded using a patch-clamp amplifier (Axopatch 200 B), the Axon-CNS (Digidata 1440 A) and Clampex software (Molecular Devices). Data were filtered at 1 kHz with low-pass filter, and stored continuously on a computer hard disc and were analysed using PCLAMP software. In some cases, the baseline was corrected with the software. Membrane voltage was clamped to −70 mV, and the sampling rate was 4 kHz. PcTx1 and APETx2 were purchased from Smartox biotechnology.

### Measurement of intracellular Ca^2+^ concentration

For cell fluorescence measurements, GSC lines were grown on glass coverslips mounted in a cell chamber and perfused with bath solution at room temperature. Fluorescence was measured continuously on an inverted microscope (IX71, Olympus, Chromaphor) using a Fluar 20 ×/0.75 objective (Olympus) and Till Vision real-time imaging software (Till Photonics). Cells were loaded for 15 min at 37 °C with 2 μM Fura-2-AM (Molecular Probes) in bath solution. Fura-2 was excited at 340/380 nm, and the emission was recorded between 470 and 550 nm using a sensicam CCD camera (PCO imaging). Acquisition and data analysis were done using Till Vision software. Ionomycin, capsaicin, menthol and ATP were purchased from Sigma-Aldrich.

### Western blot

GSC lines were lysed with RIPA buffer (25 mM Tris-Cl pH 7.6, 0.1% SDS, 150 mM NaCl, 1% Triton-X-100, 1% sodium deoxycholate, 1% PMSF, and 1% proteinase inhibitor cocktail; Roche). Samples were quantified using a protein assay (Micro BCA; Thermo Fisher Scientific) and the same amount of protein was separated using SDS-PAGE (10%). Proteins were transferred to PVDF membranes (Roche, Mannheim, Germany), and probed overnight at 4 °C with the following primary antibodies: mouse monoclonal anti-ASIC1 (NeuroMab), rabbit polyclonal anti-ASIC3 (Abcam), or mouse monoclonal anti-actin (Sigma-Aldrich.). Blots were visualized using secondary HRP-conjugated anti-rabbit or anti-mouse antibodies and SuperSignal ELISA Femto Substrate (Thermo Fisher Scientific).

### Microarray analysis

We used the publically available REpository for Molecular BRAin Neoplasia DaTa (REMBRANDT, http://www.betastasis.com/glioma/rembrandt/)^37^ to investigate expression level and prognostic significance of *ACCN2* and *ACCN3* expression. REMBRANDT is based on a total of 524 microarrays (Affymetrix U133 2.0. plus), of which 329 are included in the online database. The database was accessed September 4 2017. Data shown have been generated using the analysis tools provided at the REMBRANDT website. For Kaplan-Meyer analysis, we set the median of *ACCN* expression as threshold, such that half of the samples were defined as “low expression” and the other half as “high expression”. Statistical analysis was done with a log-rank test available at the REMBRANDT website.

### Statistical analysis

Data are reported as mean ± s.e.m. Student’s t-test was used for paired or unpaired samples. P ≤ 0.05 was considered as significant. Proton-response curves were fit with a Hill function:

$$I(x)=a+(({I}_{max}-a)/(1+{({x}_{half}/x)}^{h})$$

where *a* represents the baseline current and *I*_*max*_ the maximal normalized current, *x*_*half*_ is the concentration to elicit a half-maximal effect, and *h* represents the Hill coefficient. Expression level of *ACCN* genes in tumour and control samples was analysed by a two-sided t-test assuming equal variance with Bonferroni correction, using SPSS Statistics 24 (IBM).

### Data availability

All data generated or analysed during this study are included in this published article, except the datasets on expression levels and prognostic significance of *ACCN2* and *ACCN3* expression in patients, which are available in the REMBRANDT repository, http://www.betastasis.com/glioma/rembrandt/.
